# Investigation of the Morphology of Adrenal Glands in Hens Kept in Two Different Housing Systems—A Pilot Study

**DOI:** 10.3390/ani11072124

**Published:** 2021-07-17

**Authors:** Franziska Keßler, Angelika Grümpel-Schlüter, Christian Looft, Stefanie Petow

**Affiliations:** 1 Institute of Animal Welfare and Animal Husbandry, Friedrich-Loeffler-Institut, 29223 Celle, Germany; franziska.kessler@uni-hohenheim.de; 2 Institute of Animal Nutrition, Friedrich-Loeffler-Institut, 38116 Braunschweig, Germany; Angelika.Gruempel-Schlueter@fli.de; 3 Department of Animal Breeding and Husbandry, University of Applied Science Neubrandenburg, 17033 Neubrandenburg, Germany; looft@hs-nb.de

**Keywords:** corticosterone, interrenal cells, chronic stress, animal welfare, hyperplasia

## Abstract

**Simple Summary:**

One of the most important aspects in keeping farm animals is the housing condition, which has to fulfill the behavioral needs of the housed animals and must be economically efficient at the same time. In non-European countries, laying hens are often kept in enriched cages, while in European countries single cages are only used for breeding to record the laying performances. In contrast, floor housing pens are predominant in Germany. We assume that laying hens in single cages suffer more from chronic stress than laying hens in floor pens. To test this hypothesis, we studied the effect of housing conditions on the adrenal gland, which consists mainly of adrenal cells and interrenal cells. While the adrenal cells secrete catecholamines such as adrenalin and noradrenalin along with other hormones, the interrenal cells release steroid hormones. These also include corticosterone, which also plays an important role in chronic stress. In our study, we histologically investigated whether there is a difference in the amount of these cell types between laying hens kept in single cages and floor pens. We have found a slight tendency of a higher adrenal–interrenal ratio in floor-housed hens which is expressed in a relatively lower total area of interrenal cells in floor-housed hens. In addition, the animals in the floor housing were significantly heavier than those in the cage housing during the entire test period.

**Abstract:**

It is difficult to objectively assess the chronic effects of housing systems on livestock and particularly on laying hens. However, this seems to be important in the context of animal welfare. Therefore, we conducted the present study in order to compare the effect of two different housing conditions, single cage (SC) and floor pen (FP), on the morphology of the adrenal gland. A higher amount of interrenal cells, which secrete stress hormones, can lead to a difference in the relation of adrenal and interrenal cells, which could be interpreted as an indication of chronic stress. For this purpose, adrenal glands were extracted, prepared, stained and examined by microscopy, and total area of the cut, total area of interrenal cells and total area of adrenal cells were measured. As a result, all laying hens had a higher percentage of interrenal cells than adrenal cells (FP: interrenal cells/adrenal cells = 78.37%/21.63%; SC: 80.00%/20.00%). The median of adrenal–interrenal ratio did not differ significantly (FP = 0.2503, SC = 0.2499), while the variation of the ratio between laying hens in FP and SC showed a slight tendency of a higher ratio in adrenal glands of FP (*p* < 0.0870). Body weight and adrenal–interrenal ratio were significantly negatively correlated in laying hens in FP (r_S_ = −0.943, *p* < 0.0048) but not in SC (r_S_ = −0.162, *p* = 0.7283). There was no significant correlation between body weight and total cell area for interrenal cells or adrenal cells. Body weight was significantly lower for laying hens kept in SC than for laying hens kept in FP (*p* < 0.0001). Due to the present results, it can be concluded that keeping laying hens in single cages can have a negative effect on body weight.

## 1. Introduction

There are more than 7.7 billion laying hens kept worldwide [1]. Ninety percent of them are living in cages [1], even though there are also alternative housing conditions, such as free range and floor housing system, which is common in Germany. Laying hens that live in cages with less space and without the possibility to fulfill their needs of movement and their native behavior (e.g., dustbathing to remove lipids [2]) commonly encounter stress. Stress is a factor influencing the endocrine system [3]. Changes in the morphology of the adrenal gland, particularly hyperplasia of the interrenal cells, enlargement of the whole adrenal gland and increased adrenocortical activity could be a hint of chronic stress [4,5].

The adrenal gland consists of blood vessels and adrenal and interrenal cells [6]. In contrast to adrenal glands in mammals, adrenal and interrenal cells are located side by side in the adrenal glands of birds [7]. Adrenal cells secrete hormones, e.g., adrenalin and noradrenalin; interrenal cells secrete corticosteroids such as corticosterone [8].

It is already known that corticosterone is a hormone that increases blood glucose levels and stimulates gluconeogenesis [7] during a stress response and is essential for the organism in general, e.g., immunosuppression or regulation of blood sugar.

Along the hypothalamus–pituitary axis (HPA), corticotropin-releasing hormone (CRH) is first released in the hypothalamus, when an organism gets an impulse. The CRH then causes the release of adrenocorticotropin (ACTH) in the pituitary gland [8], which results in the secretion of corticosterone [7,9].

A chronic release of ACTH causes chronic stimulation of the adrenal gland. This can lead to hyperplasia of the interrenal tissue in the adrenal gland based on the fact that the corticosterone secretion rate increased [4]. Consequently, secretion of corticosterone and its effects on the adrenal gland are parameters of how much an animal suffers from chronic stress [5].

There are different parameters used to compare the effects of housing conditions on animal health, such as feather score [10] or bone strength [11], but so far there is no objective method available to measure the influence of chronic stress. Former studies analyzed the concentration of corticosterone in plasma or eggs, which are suitable to assess a current but not a chronic stress load (e.g., [12]). Comparing the area of cells in the adrenal gland of laying hens treated by dexamethasone, formalin or both substances showed significant differences in the development of cells in the adrenal gland [4]. Here, we used a similar method to compare the influence of two different housing systems on the morphology of the adrenal gland in laying hens.

This study investigated the following hypotheses: Firstly, we suppose that laying hens in single cage (SC) housing condition chronically suffer from more stress than laying hens in floor pen (FP) housing condition, because the SC housing condition differs more from the natural environment than the FP housing condition. Secondly, as a consequence of the first hypothesis, the ratio of adrenal–interrenal cells is lower in SC, because hens in this housing condition suffer from more stress and need a higher percentage of interrenal cells to secrete a higher amount of corticosterone. Our third hypothesis is that laying hens in SC could show a hyperplasia of their adrenal cells in relation to the body weight, because we suppose an abnormal development due to a constant high corticosterone secretion.

## 2. Materials and Methods

The present study was performed in accordance with the German Animal Protection Law and was approved by the Lower Saxony State Office for Consumer Protection and Food Safety (No. 33.9-42.502-05-10A079).

### 2.1. Animals and Housing Conditions

Fourteen WLA laying hens (Lohmann Tierzucht, Cuxhaven, Germany), which descend from a commercial breeding program, were used for this investigation. The chickens hatched on the same day and were raised in a floor housing system. The stables were 6 m × 4 m; littered with a mix of straw and wood shavings; and equipped with a feeding trough, two drinking nipples and perches.

During rearing phase, the animals had ad libitum access to food (weeks 1–7: 12.97 MJ AME_N_/kg DM, 189.61 g/kg crude protein, 31.38 g/kg crude fat, 9.14 g/kg Ca, 6.94 g/kg P; weeks 8–16: 12.82 MJ AME_N_/kg DM, 151.67 g/kg crude protein, 30.21 g/kg crude fat, 15.83 g/kg Ca, 8.11 g/kg P) and water. The first 48 h after hatching, light was provided 24 h/day. On the third day, light was reduced to 15 h per day and consecutively weekly reduced to 9 h per day until week 7. From week 7 until week 16, light was provided 9 h per day, which corresponds to the light schedule in conventional practice.

After rearing until their 16th week of age and before the start of the experiment, laying hens were randomly divided into a floor housing system (FP) or a single cage system (SC). The SC had the size of 50 cm × 46 cm × 43 cm. It contained a feeding trough, two drinking nipples and a perch. The floor pens were also equipped with feeding troughs and drinking nipples and had a size of 8 m^2^ (2 m × 4 m). On a slatted floor 0.5 m above the ground were perches in front of nests. The floor pens were littered with wood shavings.

The SC and the FP were located in the same stable, and thus laying hens in both systems were exposed to the same climatic conditions. Light phase was 16 h per day. In addition to the 7 animals used for this study, 21 other animals were kept alongside in FP for another study.

All animals had ad libitum access to food (11.68 MJ AME_N_/kg DM, 168.11 g/kg crude protein, 29.43 g/kg crude fat, 50.05 g/kg Ca, 5.06 g/kg P) and water.

### 2.2. Data Collection

At the end of the experiment, we excluded the data of one laying hen of the FP group. It showed a constant decrease in body weight until the end of the experiment. Thus, we analyzed seven laying hens kept in SC and six laying hens kept in FP.

The animals were weighed once a month from their 17th week of life until the end of the experiment at their 75th week of life.

Then, all animals were anesthetized with isoflurane and killed by bleeding. Both adrenal glands were extracted and fixed in 4% formaldehyde (Formalin, RotiHistofix, Roth, Karlsruhe, Germany). After 48 h, the adrenal glands were dehydrated with denatured ethanol and xylol (both Roth, Karlsruhe, Germany) and embedded in paraffin wax (Paraplast PLUS, Roth, Karlsruhe, Germany). The fixed organs were cooled at 4 °C until cutting. Subsequently, the adrenal glands were cut into 4 μm thin sections using a microtome (Leica, Wetzlar, Germany). Every 5th section was fixed onto a covered slide with a mixture of protein and glycerin (both Roth, Karlsruhe, Germany) and dried in an incubator at a temperature of 54 °C for an hour afterward. Afterward, the slices were stained with hematoxylin and eosin (Roth, Karlsruhe, Germany).

### 2.3. Microscopical Analysis

**Figure 1:**
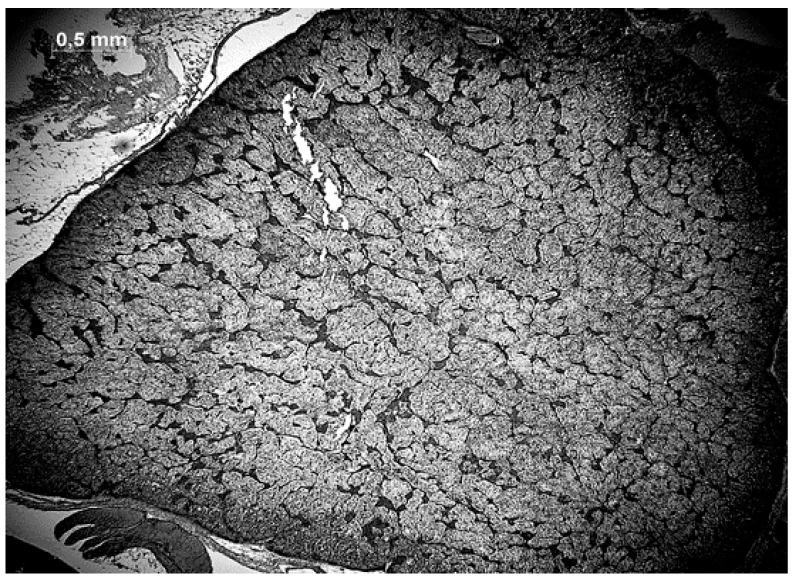
Adrenal gland with adrenal cells (dark grey areas) and interrenal cells (light grey areas).

**Figure 2:**
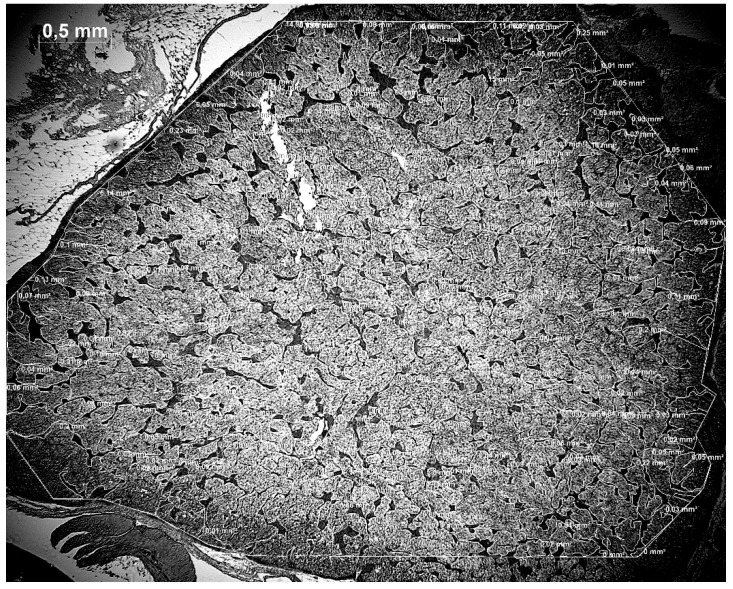
Adrenal gland with encircled and measured total area without artifacts, blood vessels and nonidentifiable tissues but with encircled and measured interrenal cells.

For microscopical analysis, five sections of each adrenal gland (Figure 1) were examined under a microscope (Stemi 305, Zeiss, Jena, Germany) and photographed (Axiocam 506 mono, Zeiss, Jena, Germany) with an amplification of 25 fold (N = 130). The evaluation of the size area was made using the ZEN software (ZEN 2.1 black; Version 11.0.0.190; ZEISS; Jena, Germany). As a first step, the total area was defined and artifacts, blood vessels and unidentifiable tissue were excluded. Then, the area of interrenal cell parts was circled and the given interrenal area was subtracted from the total area to determine the adrenal cell parts (Figure 2).

### 2.4. Statistical Analysis

Body weight was tested for statistical differences using a two-way ANOVA (proc mixed in SAS 9.4 [13]) with housing system, week of age and interaction of week of age and housing system as fixed factors. Body weight at the 17th week of age was included as a covariable to compensate for weight differences of the animals at the beginning of the study in the analysis.

The adrenal–interrenal ratio was built by dividing the area of adrenal cells by the area of interrenal cells. The influence of the housing system on the relation of adrenal and interrenal cells in adrenal glands was analyzed using a linear mixed model (PROC GLIMMIX in SAS 9.4). The housing condition (single cage/floor pen) was tested as the fixed effect; the adrenal–interrenal ratio was taken as the dependent variable. The number of pictures per laying hen (right/left; Section 1, Section 2, Section 3, Section 4 and Section 5) was involved as a repeated measurement. The Gaussian distribution was tested using the Shapiro–Wilk test.

In order to calculate the Spearman correlation coefficient between body weight and total area of adrenal or interrenal cells, as well as between adrenal–interrenal ratio and body weight (PROC CORR in SAS 9.4), the median area of adrenal and interrenal cells and the adrenal–interrenal ratio were calculated to account for the measurement repetitions resulting from multiple cuts per animal. The significance level was set by *p* < 0.05.

## 3. Results

### 3.1. Development Body Weight

**Figure 3:**
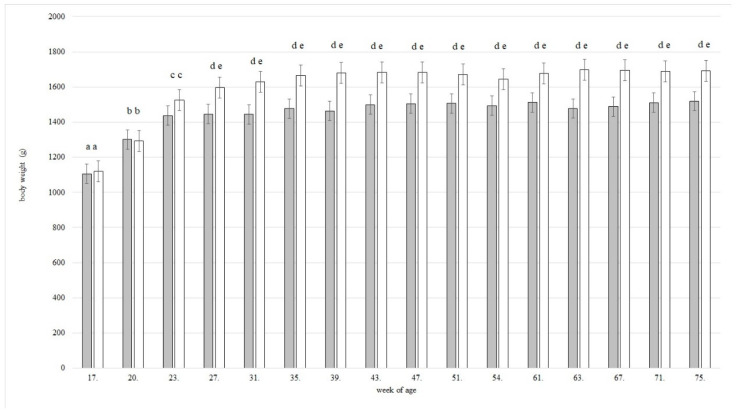
Development of body weight during the whole study (*n* = 7 laying hens in single cages, grey bars; *n* = 6 laying hens in floor pens, white bars). Figure shows means of least squares plus/minus standard error. Lowercase letters indicate significant differences (*p* < 0.05).

The body weight of laying hens in both groups increased from week 17, the start of the experiment, to their 27th week of age (Figure 3). From week 27, the laying hens in both groups reached a plateau and maintained their weight. Body weight differed significantly between 17th, 20th and 23rd weeks of age in both groups (*p* < 0.0001). From week 27, laying hens in FP were significantly heavier than laying hens in SC (*p* < 0.0001). There were no significant differences between the groups during the first three weeks of the experiment.

### 3.2. Total Area of Cells in Adrenal Gland and Adrenal–Interrenal Ratio

The median of total area of adrenal cells varied slightly in laying hens in SC (median = 2.236 mm^2^, min = 1.189 mm^2^, max = 4.260 mm^2^) and FP (2.415 mm^2^, 1.375 mm^2^, 3.790 mm^2^). Adrenal cells represented 20.00% of the total area in laying hens in SC. Laying hens in FP had 21.63% adrenal cells.

The median of total area of interrenal cells did not differ significantly between SC (9.168 mm^2^, 6.200 mm^2^, 13.227 mm^2^) and FP (8.225 mm^2^, 5.216 mm^2^, 14.626 mm^2^). Laying hens in SC had 80.00% interrenal cells, and laying hens in FP had 78.37% interrenal cells.

The adrenal–interrenal ratio showed the relation between the total area of adrenal cells and the total area of interrenal cells. If the ratio was <1, the area of interrenal cells was larger than the area of adrenal cells; if the ratio was >1, the area of interrenal cells was smaller than the area of adrenal cells.

**Figure 4:**
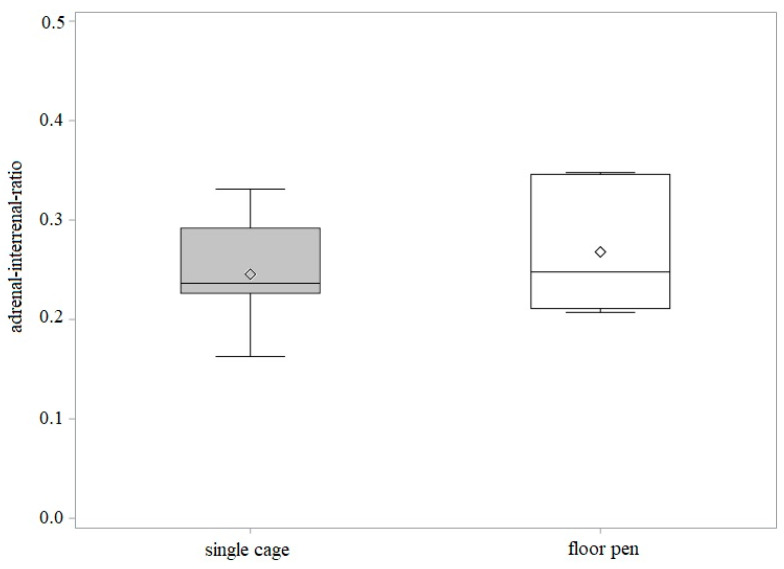
Adrenal–interrenal ratio in single cages (*n* = 7 laying hens, grey box) and in floor pen (*n* = 6 laying hens, white box). The vertical boxes show the range between the 25th and 75th percentiles with whiskers extending to 2.5 of the interquartile range. The horizontal line indicates the median; diamond indicates the mean.

The median ratio did not show a significant difference between laying hens in FP (0.2503) and laying hens in SC (0.2499; Figure 4), while the variation of the ratio between laying hens in FP and SC showed a slight tendency of a higher ratio in adrenal glands of the FP group (*p* < 0.0870).

### 3.3. Correlation between Body Weight and Cells of the Adrenal Gland

**Figure 5:**
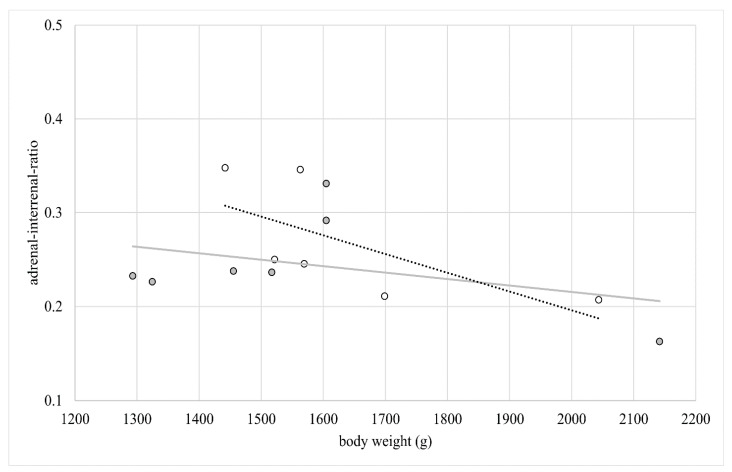
Correlation between body weight and adrenal–interrenal ratio (*n* = 7 laying hens in single cages, grey dots; *n* = 6 laying hens in floor pens, white dots) Lines show the linear correlation between body weight and adrenal–interrenal ratio for SC (grey) and FP (black dotted).

Neither the area of interrenal cells nor the area of adrenal cells showed a significant correlation with the body weight in SC or FP. There was a strong negative correlation of the body weight with adrenal–interrenal ratio in the FP group (r_S_ = −0.943, *p* < 0.0048) but not in the SC group (r_S_ = −0.162, *p* = 0.7283; Figure 5).

## 4. Discussion

As outlined in the introduction, the aim of this present study was to investigate the influence of the environment on the morphology of the adrenal glands. We supposed that laying hens in SC suffer from more stress than laying hens in FP. This hypothesis is based on previous studies that compared different housing conditions. Researchers often found better animal health parameters in laying hens living in floor pens than in those living in single cages, e.g., higher bone strength [10,14,15], lower percentage of osteoporosis [16], fewer foot and toe lesions [10,17], better feather condition [10,17] and lower risk of fatty liver [16,18], and even found that furnished cages cause better animal health, e.g., fewer foot lesions [19,20] and better claw health [19]. Observed advantages of single cages were a smaller number of fractures [16] and a lower susceptibility to illness due to a possibly higher level of hygiene [16,19]. Furthermore, laying hens have the need to behave naturally and interact with conspecifics [19]. They want to use perches [12,21,22] and stretch their extremities [23] and need to dust bathe up to 5% of the daily time [12,21]. Otherwise, they show the abnormal behavior of sham dust-bathing [24] and cannot remove lipids on their feathers [2,24]. In comparison to laying hens in enriched cages and in floor pens, laying hens in conventional cages show abnormal behavior of aggressive pecking at their cages [12,21,23], and laying hens in group cages exhibit abnormal aggressive pecking at their conspecifics [21,25]. Thus, conventional cages can have a negative impact on animal health (e.g., bone strength, foot lesions, feather condition) and animal welfare (aggression against conspecifics; missing possibilities to perch, dust bathe or stretch) [19], which is why we assumed that the single cage housing parameters acted as stressors per se. Under consideration of all these stressors, we supposed an effect on cells in the adrenal gland as Tapan et al. found in 1972 by inducing stress with dexamethasone and formalin [4]. In our study, we could not find such an effect; the medians of adrenal–interrenal ratio did not differ significantly.

We expected that laying hens in single cage (SC) housing condition should have a lower adrenal–interrenal ratio than laying hens in floor pen (FP) housing condition because of the increased corticosterone secretion due to chronic stress. This could lead to more interrenal cells. We could not prove such an effect in the present study. There was no significant difference between median adrenal–interrenal ratios of laying hens kept in FP and in SC. However, there was a slight tendency of a higher ratio in adrenal glands of the FP group in variation.

The third hypothesis, namely that laying hens in SC should show hyperplasia of their adrenal cells in relation to the body weight, could not be confirmed. Our hypothesis was based on another study, where turkeys showed hyperplasia after forced training [26]. In the present study, the body weight was not correlated with the area of the adrenal gland cells. We found that the body weight correlated strongly negatively with the adrenal–interrenal ratio of the laying hens in FP. This was a surprising result as we expected to find a positive correlation between body weight and adrenal–interrenal ratio in FP.

### 4.1. Development of Body Weight

Laying hens in FP were significantly heavier than their counterparts in SC. These results confirmed earlier studies, e.g., Knowles et al., 1990 [14]; Küçükyılmaz et al., 2012 [27]; and Dikmen et al., 2016 [10]. The heavier animals in FP could be due to a better physical condition as a consequence of the possibility of higher physical activity. Knowles and Broom (1990) described a positive correlation between body weight and bone strength in laying hens housed in terraces and percheries [14]. Furthermore, broilers with a higher activity level had a higher body weight [28] due to a higher muscle weight. As shown in previous studies [12,29], the body weight could be an indicator of chronic stress. In our study, the body weight was significantly lower in the SC group (*p* < 0.0001). This result could therefore be interpreted as indicative of hens kept in SC suffering more from chronic stress than the hens kept in FP because they could not meet their behavioral needs and had no direct contact with their conspecifics. However, we could not track the food consumption in FP compared to SC. Therefore, it remains unclear whether the animals took in less food in relation to hens in FP. It could also be that the animals in SC consumed the same amount of food but did not anabolize it due to a higher stress level or less activity. However, as generally known, less activity could lead to a weight gain due to too little movement. We therefore assume that the lower body weight was an indicator of chronic stress in SC.

### 4.2. Total Area of Cells in Adrenal Gland and Adrenal–Interrenal Ratio

Total areas of cells in the right and left adrenal glands were considered together because both sides experienced the same effects.

All laying hens had a higher proportion of interrenal cells than adrenal cells, regardless of their housing condition. Laying hens in FP had more adrenal cells than laying hens in SC, and thus laying hens in SC had more interrenal cells than laying hens in FP, which corresponds to other studies, which also describe a higher percentage of interrenal cells in laying hens [6]. Ratios vary between 33% adrenal cells, 52% interrenal cells and 15% blood vessels [30] and 20–30% adrenal and 70–80% interrenal cells [8] in laying hens.

The median of the adrenal–interrenal ratio did not show a significant difference between laying hens in FP and laying hens in SC. The variation of the ratio showed a slight tendency of a higher ratio in adrenal glands of laying hens in FP.

In view of these results, the hypothesis that laying hens in SC have a higher proportion of corticosterone-secreting interrenal cells than laying hens in FP could not be confirmed.

We assumed that laying hens in SC suffer more stress than laying hens in FP. Since the laying hens in both groups were kept in the same stable, received the same feed and had the same light regime, the effects of housing in floor pens or individual cages were the main factors that differed between the groups. The general mechanism of a stress response is the secretion of ACTH in the pituitary [8], which leads to the release of corticosterone in the adrenal gland in interrenal cells [7,9,31,32]. A repeated release of ACTH in high distribution as a consequence of a chronic stressor and a subsequently increased concentration of corticosterone could cause hyperplasia of the adrenal gland [33,34]. In chronically stressed laying hens, there could be a shift in the adrenal–interrenal ratio.

Since the adrenal interrenal ratio did not differ between laying hens in SC and in FP, it cannot be concluded that the animals in one group were more stressed than the laying hens in the other.

Consequently, the second hypothesis that laying hens in conventional cages suffer more from stress than laying hens in floor pens cannot be confirmed.

### 4.3. Correlation between Body Weight and Cells of Adrenal Gland

There was no significant correlation between body weight and total cell area, either from adrenal or interrenal cells.

Body weight and adrenal–interrenal ratio showed a strongly negative correlation in FP laying hens, whereas in SC hens, the correlation was also negative, but not significant. A negative correlation means that the adrenal–interrenal ratio decreased and the body weight increased. Thus, the area of interrenal cells increased with an increasing body weight. This development was already described in turkeys after forced training [26].

In the present study, we did not collect any individual animal health or animal welfare parameters, so it is not possible to identify clear factors that explain our correlation.

As in comparable studies [4,26], we wanted to prove hyperplasia, which required an adrenal weight. Therefore, the third hypothesis, that laying hens in single cages suffer from adrenal hyperplasia, can neither be confirmed nor rejected at this time. It is important to measure adrenal gland weight in the following studies.

## 5. Conclusions

All of the laying hens in the present study showed a greater total area of interrenal cells than adrenal cells.

There was no significant difference in the median adrenal interrenal ratio between laying hens kept in SC and FP, meaning that the relative area of interrenal cells in the adrenal gland did not differ between the groups.

Body weight did not correlate positively with total cell area in the adrenal gland, neither with interrenal cells nor with adrenal cells.

Therefore, the present study could not support our hypothesis that laying hens in SC single cages suffer from more stress than laying hens in FP. The morphology of the adrenal gland did not differ between the groups.

Since we found significant differences in body weight, this could be a simple indicator to record a possible negative influence of the housing system.

## Data Availability

The data presented in this study are available on reasonable request from the corresponding author.
